# An Open Source Low-Cost Device Coupled with an Adaptative Time-Lag Time-Series Linear Forecasting Modeling for Apple Trentino (Italy) Precision Irrigation

**DOI:** 10.3390/s21082656

**Published:** 2021-04-09

**Authors:** Simone Figorilli, Federico Pallottino, Giacomo Colle, Daniele Spada, Claudio Beni, Francesco Tocci, Simone Vasta, Francesca Antonucci, Mauro Pagano, Marco Fedrizzi, Corrado Costa

**Affiliations:** 1 Consiglio per la Ricerca in Agricoltura e L’analisi Dell’economia Agraria (CREA)—Centro di Ricerca Ingegneria e Trasformazioni Agroalimentari—Via della Pascolare 16, 00015 Monterotondo, Rome, Italy; simone.figorilli@crea.gov.it (S.F.); claudio.beni@crea.gov.it (C.B.); francesco.tocci@crea.gov.it (F.T.); simone.vasta@crea.gov.it (S.V.); francesca.antonucci@crea.gov.it (F.A.); mauro.pagano@crea.gov.it (M.P.); marco.fedrizzi@crea.gov.it (M.F.); 2 Effetreseizero Srl, Spinoff CREA, Via dei Solteri 37/1, 38121 Trento, Italy; giacomo.colle@f360.it (G.C.); daniele.spada@f360.it (D.S.)

**Keywords:** digital agriculture, precision agriculture, IoT, Arduino, LoRa, adaptative modeling, DSS decision support system

## Abstract

Precision irrigation represents those strategies aiming to feed the plant needs following the soil’s spatial and temporal characteristics. Such a differential irrigation requires a different approach and equipment with regard to conventional irrigation to reduce the environmental impact and the resources use while maximizing the production and thus profitability. This study described the development of an open source soil moisture LoRa (long-range) device and analysis of the data collected and updated directly in the field (i.e., weather station and ground sensor). The work produced adaptive supervised predictive models to optimize the management of agricultural precision irrigation practices and for an effective calibration of other agronomic interventions. These approaches are defined as adaptive because they self-learn with the acquisition of new data, updating the on-the-go model over time. The location chosen for the experimental setup is a cultivated area in the municipality of Tenna (Trentino, Alto Adige region, Italy), and the experiment was conducted on two different apple varieties during summer 2019. The adaptative partial least squares time-lag time-series modeling, in operative field conditions, was a posteriori applied in the consortium for 78 days during the dry season, producing total savings of 255 mm of irrigated water and 44,000 kW of electricity, equal to 10.82%.

## 1. Introduction

Traditional irrigation consumes great amounts of water and electrical energy because it applies water uniformly over every part of the field without considering the variability of soil and crop different water needs. Therefore, following the soil structure and the vegetation, some parts of the field often result to be over-irrigated, while other parts are under-irrigated [1].

As opposed to uniform irrigation, precision irrigation involves the principle of variable rate applications based on plant needs and environmental conditions to ensure optimal production with minimal impact and resource use [2,3].

Generally, the development of modern society is associated with population growth and the consequent necessity to increase the production of world agricultural food. As reported by Alt et al. [4], this means that agricultural production processes should become more efficient, and there is an inevitability of digitalization of all agricultural systems. This is made possible using intelligent technologies (e.g., artificial intelligence, robotics, Internet of things (IoT), unmanned aerial vehicles, etc.), which could increase productivity, reduce production costs, and reduce labor requirements [5].

Generally, in smart irrigation practices, the application of sensors and networking units represents an efficient solution to handle the limitedness of essential resources such as water and consequently increase crop yields. The sensors suitable for this purpose are primarily meant to be operated remotely taking advantage of IoT ecosystems while taking precise measurements relevant for irrigation planning such as the amount of water, crop temperature, and humidity to build a robust supply chain ecosystem and make decisions [6].

To assess the impact of irrigation heterogeneity on crop yield and soil water management practices, some studies integrated forecasting modeling approaches to provide innovative frameworks and predict agronomic and economic impacts. An example is a work carried out by Bellvert et al. [7], which developed a model for scheduling irrigation simulating the actual amount of water evapotranspirated per vine to assess the necessary amount of water to be applied when different irrigation strategies were adopted. The study of Xu et al. [8] developed a decision model interfacing data relative to agricultural production, crop characteristics, and irrigation regularity implemented online for remote web services.

The water balance method balancing water inputs and outputs of the soil–plant system is widely used to schedule irrigation in arboriculture [9]. The soil–plant water need is constituted by crop evapotranspiration (ETc), which is obtained by multiplying evapotranspiration of the reference crop (ETo) with the crop coefficient (Kc) as a function between soil–plant system water needs and climate conditions [10,11].

The description of the soil’s water fluxes at spatial and temporal scale is required to simulate complex processes regarding plant (i.e., growth, root uptake and assimilates translocation), soil (i.e., infiltration, runoff, and percolation), groundwater (i.e., capillary rise and water table dynamic), atmosphere (i.e., evaporation and air fluxes), and crop practices (i.e., soil management and planting density) [11,12,13].

In light of the literature, the present study aims at the creation of a highly accurate adaptive multivariate modeling and the development of a decision support system (DSS) based on a mobile app, open source IoT LoRa weather station, and ground sensors automatically updating field measurements. Finally, the integration of the obtained adaptive supervised predictive models allowed optimal precision irrigation scheduling, therefore calibrating effectively other agronomic interventions. This approach is defined as “adaptive” because it self-learns with the acquisition of new data, updating the model on the go over time.

## 2. Materials and Methods

### 2.1. Experimental Field and Setup

The location chosen for the experimental setup is a cultivated area in the municipality of Tenna, Trentino, Alto Adige region, Italy. The area is about 50 hectares, located in a mountainous area at 569 m a.s.l., and mainly dedicated to the production of apples and berries.

Tenna is characterized by a warm and temperate climate with high rainfall throughout the year, including the driest month. According to Köppen and Geiger [14], the climate is classified as oceanic temperate (Cfb), the average temperature is 10.4 °C, and the average annual rainfall is 862 mm.

The difference in precipitation is 56 mm between the driest month and the wettest month. Average temperatures vary by 21.3 °C over the course of the year (Table 1).

In recent decades, the area has been characterized by climate change; Table 2 shows the average monthly temperatures of 2019, which highlights sharply increased values.

The irrigation of the area is managed by a local consortium of small agricultural producers, the Consorzio di Miglioramento Fondiario di Tenna.

**Figure 1:**
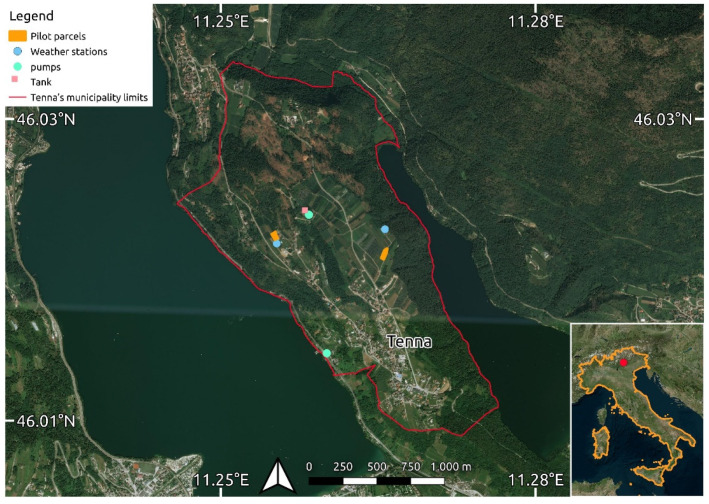
An experimental area of the project: Tenna, Trentino, Alto Adige, Italy.

The experimental setup consists of two experimental parcels in the Tenna area (Figure 1).

The east parcel, with an area of 0.21 ha, is cultivated with golden apples on rootstock M9, planted in 2015, with a planting distance of 0.80 × 3.00 m. The west parcel, with an area of 0.17 ha, is cultivated with Crimson Crisp apples on M9 rootstock, planted in 2014, with a planting distance of 0.80 × 3.50 m. Apples begin the vegetative cycle around March 20th. For both varieties, the fruits are harvested in the last week of September, and leaf fall occurs in the first days of November.

Both parcels are managed through driplines irrigation systems. The water is collected from the Caldonazzo lake at a quota different by 187 m. The difference in altitude is surmounted by means of an electrical 75 kW pump with a flow of 26 L m^−1^. At the top of the pumping system, a cistern of 200 m^3^ collects the water pumped from the lake and, from there, drops by gravity to the parcels. Two other 9 kW parallels pumps, with a flow of 18 L m^−1^, guarantee the water supply to the upper fields of the consortium.

The conventional irrigation scheduling adopted for the entire consortium’s parcels is equal to 1 h a day with a water volume of 5.25 mm m^−2^. The west experimental parcel was used as control for the irrigation model comparison, being managed through conventional irrigation strategy.

Both soil parcels are characterized by sandy loam texture in the first 90 cm of depth. The organic matter (OM) content in T1 (east parcel) is considered moderately low at 30 cm and 60 cm and low at 90 cm, while in T2 (west parcel) soil OM is moderately low at 30 cm and very low (not quantifiable) at 60 cm and 90 cm. Nevertheless, in T2, high content of organic matter on the topsoil was detected. Both soils are considered noncalcareous. The available water capacity (AWC) tends to be low, consistent with the sandy classification of the two soils. This would confirm the need for a specific differential irrigation strategy in terms of volumes and number of interventions. Due to the presence of skeletons (particles with a diameter >2 mm), especially in T2 (west) soil, particular attention was paid to sampling the soil.

Soil is managed with inter-row grassing and chemical weeding on the row. The root depth of apple trees, being on weak rootstocks M9, reaches a maximum of 0.40 m.

In each experimental parcel was installed a weather station and soil moisture sensors connected via LoRaWAN protocol.

The installed weather stations (Table 3) are Davis Vantage Pro2 models, engineered to handle the harshest environments and deliver data with scientific precision. It was used to collect the following data: air temperature and humidity, dew point, wind speed and direction, barometric pressure, and rainfall. The installed model is also capable of communicating data through a mobile Internet connection to the web database and it is also integrated with a LoRaWAN gateway to collect data from remote field sensors. In addition, extra sensors and accessories could be added to the Vantage Pro 2 station to allow the design of sophisticated environmental monitoring systems, building up an adapted configuration.

### 2.2. The Open Source Soil Moisture LoRa Device

**Figure 2:**
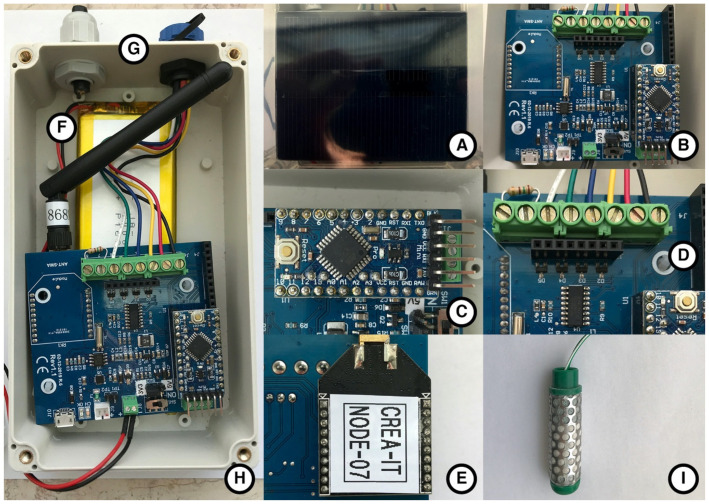
Soil moisture LoRa (long-range) device (**H**) and the solar panel needed to charge the LiPo battery (**A**). The main interface expansion shield used by the microcontroller (**B**), which is an Arduino Mini Pro (**C**) providing all the inputs and outputs pins (**D**). Below this, the electronics lies the LiPo battery (**F**) (1S, 3000 mAh). This board is equipped with a socket (XBee form factor) for the wireless hardware, LoRaWAN (long-range wide-area network), used to connect to the main gateway (LoRaWAN protocol) (**E**). In addition, a waterproof connector provides connectivity with the soil sensors (**G**). Soil moisture sensor resistant electrodes (**I**).

The soil moisture LoRa device is based on open source technology (software and hardware). Mainly, it is equipped with soil moisture sensors, based on resistive technology, which exploits the relationship principal constant between the ohmic variation and pressure given by the soil water tension, soil moisture, air quality, and rainfall height sensors. The model used is the Watermark Soil Moisture Sensors 200SS (The Irrometer Company Inc., Riverside, CA, USA), a well-established method of assessing soil moisture in crops with a good value for money. The acquired information is transferred through radio waves using LoRaWAN (long-range wide-area network) technology. The transmission protocol was chosen because it guarantees greater autonomy of the device and a range of action on several km. The whole system was optimized to reduce energy consumption, making it possible to power it through a LiPo battery recharged through a small solar panel (Figure 2).

The device was designed in a single electronic board (Figure 2), integrating different numbers and typologies of sensors. This device can integrate many types of sensors, for example, those typical of a weather station (e.g., rain gauge and air quality), monitor the status of the battery and solar panel, with a dedicated connection for debugging. Finally, the actual communication system, currently based on LoRaWAN, can be replaced for other applications with different technologies such as Wi-Fi and Xbee.

The selected soil moisture sensors (Figure 2) consist of a pair of highly corrosion-resistant electrodes embedded in a granular matrix. A current is applied to the sensor to obtain a resistance value. The sensor meter correlates the resistance to centibars (kilopascals) of the soil water tension. The sensor has also been designed to be a permanent sensor, positioned in the matrix to be monitored. Finally, an important feature, for the reliability of the reading, is the presence of plaster installed internally, which provides a buffer effect considering the effect of the salinity levels normally present in crops and irrigated agricultural landscapes.

The sensors installed for the experimental activities of this study are equipped with three moisture sensors (installed at 30–60–90 cm depth) and a rain gauge.

**Figure 3:**
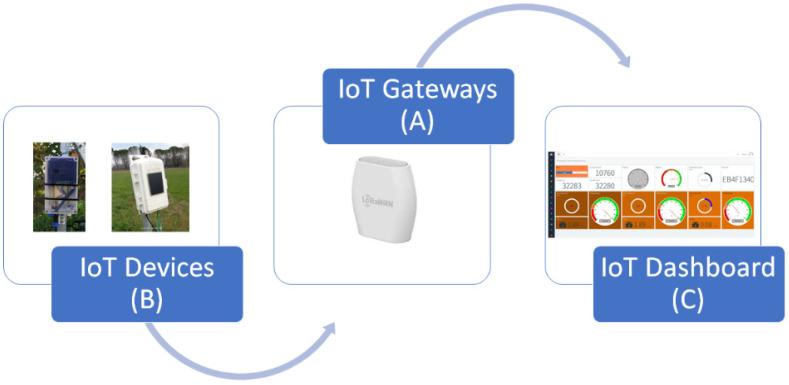
Data acquisition and historicization infrastructure: (**A**) LoRaWAN gateway for the direct connection of the sensors via LoRaWAN transmission and (**B**) IoT remote service for the creation of (**C**) the dashboard and historicization of data.

The data acquisition and historicization infrastructure is composed by the LoRaWAN gateway, for a direct connection of the sensors via LoRaWAN transmission and an IoT remote service for the creation of the dashboard (Figure 3) and historicization of data.

The device transmits the packet containing the read values to the gateway on which it has established a LoRaWAN connection. Then, the gateway forwards the packet to the remote service that will historicize and display according to the created mask. This service is accessible from any internet point. The approximate cost of the open source device with the configuration described above is around EUR 350.00, which can be considered low cost, compared to a commercial device on the market.

The developer open source device was operatively compared with the Sentek Drill & Drop soil moisture and temperature sensors installed in the Tenna parcels. These are capacitive frequency domain reflectometry (FDR) sensors with a probe length of 90 cm and a step of measurement of 10 cm in depth.

### 2.3. Data Acquisition and App

**Figure 4:**
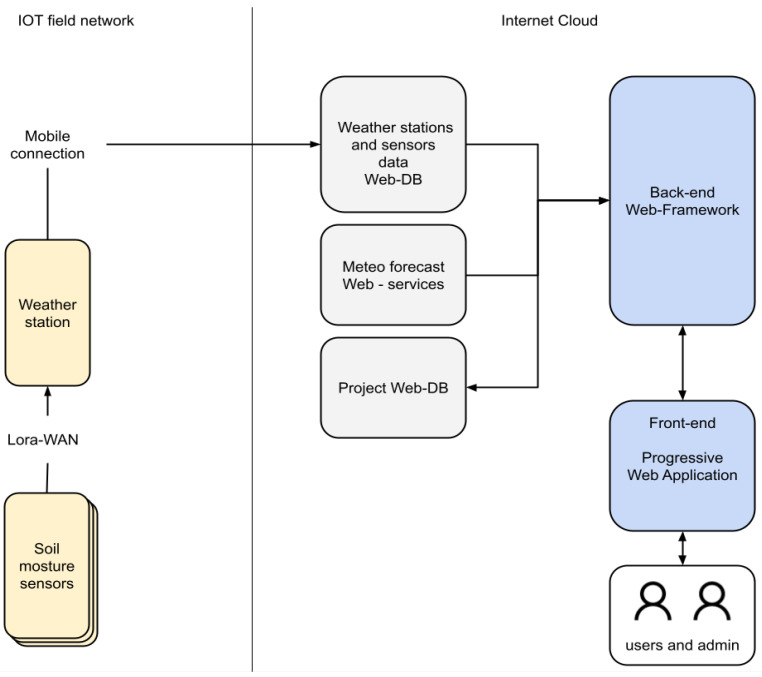
Field network and cloud components for data acquisition.

The experimental area sensors operate through an IoT LoRaWAN local network and transmit data to a web-based software framework that manages the back end of the system, the cloud databases, and the interface with final users called via a web application (Figure 4).

**Figure 5:**
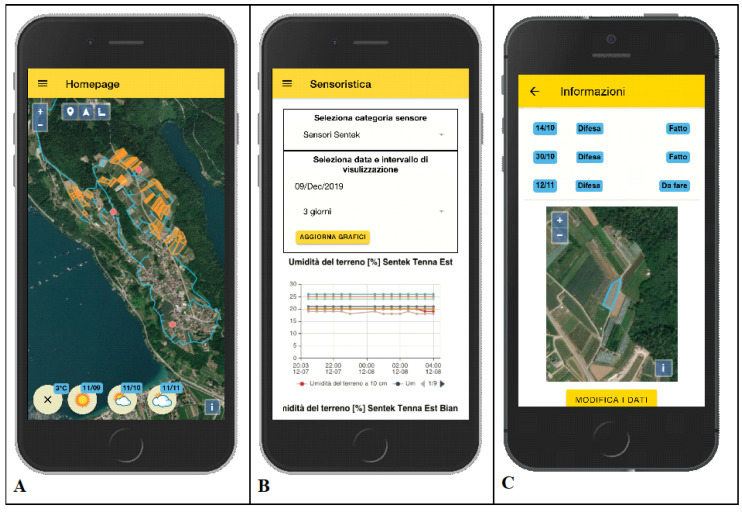
Progressive web application: (**A**) farm parcels and weather forecast, (**B**) moisture sensors data, and (**C**) agricultural activities register.

The front-end software was developed as a mobile progressive web application (PWA), and it was implemented using Ionic and Angular frameworks (Figure 5). The app is available for Android and iOS and, alternatively, it is accessible via a common browser. The app allows final users to access the data collected by sensors, manage their cultivation, the agricultural activities, and, for admin users, monitor water and energy consumption. Moreover, the web application allows admin users to check the precision irrigation model outputs and evaluate irrigation decisions.

The back-end web framework is a server-side REST API (Representational State Transfer Application Programming Interface), implemented in Loopback. It provides a common interface to the front-end application to access the different data sources of the project—sensors data, weather stations data, weather forecast data. It also provides the interface to store and retrieve in a MongoDB web-DB user data such as parcels administrative and geographical data, fields data, agricultural activities log, etc.

**Figure 6:**
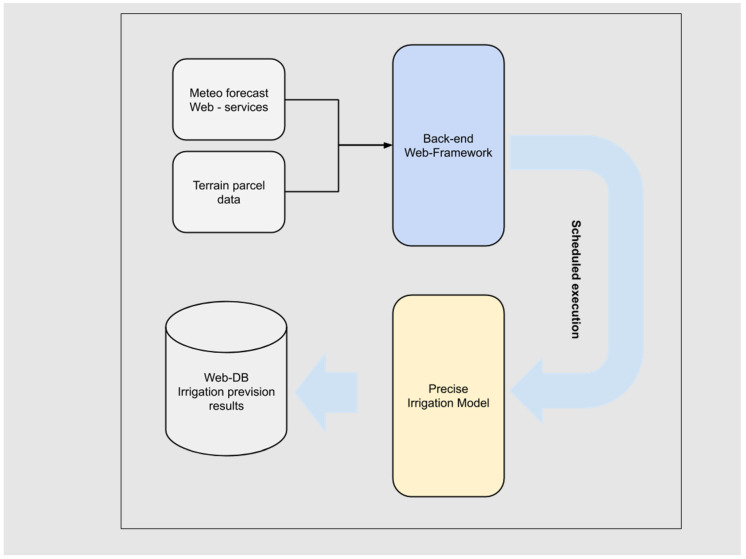
Irrigation model scheduled execution data flow.

The back-end web framework (Figure 6) has also a specific developed service to execute the predictive modeling for precise irrigation. Precise irrigation model’s software implementation consists of a MATLAB code runnable via MATLAB Runtime Compiler. The model execution needs field terrain data stored in the project web database and updated weather forecast data. A server-side Node.js application runs independently at a model-dependent scheduled time, from one time every day to one time every 6 h, collects data from weather service and database, and stores new irrigation prevision results on it; it also preserves previous predictions.

The irrigation previsions are immediately and automatically available in the mobile web application and could be used to adapt the irrigation strategies. They could also be part of the necessary data needed to pilot the automatic irrigation system.

**Figure 7:**
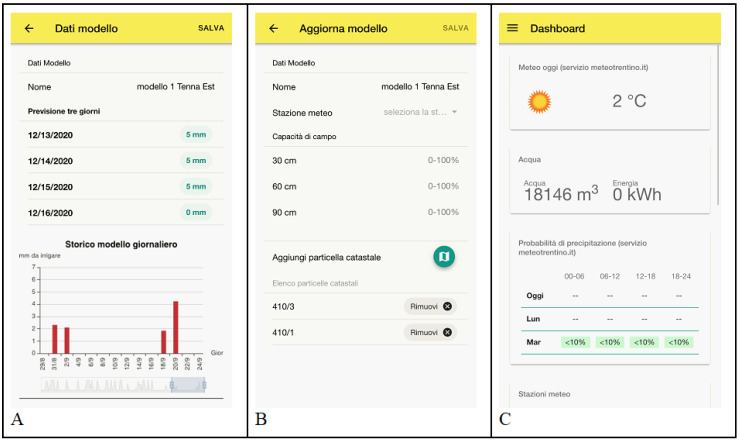
From left: model’s prevision and historical prevision data graph, model terrain settings and parcel association, and app’s dashboard with the seasonal amount of consumed water in m^3^.

In the mobile web application (Figure 7), admin users could also examine historical previsions data of the specific model and manage the association of the model with compatible parcels of the consortium. Finally, a panel in the app dashboard quickly summarizes the total amount of water used from the beginning of the irrigation season, using the predictive irrigation model instead of the classic daily constant rate.

### 2.4. Predictive Modeling

The database was constructed with data collected from the weather station and from the sensors (commercial ground sensors and open source soil moisture LoRa) distributed in the Tenna consortium from 19 June to 3 September 2019 (77 days).

The weather station collected the following parameters every 30 minutes: air humidity (%), air temperature (i.e., maximum, minimum, and average (°C), dew point temperature (°C), wind direction, mean wind speed (km∙h^−1^), gust wind speed (km∙h^−1^), rainfall height (i.e., H_2_O rain; mm), solar radiation (Watt∙m^−2^)).

**Figure 8:**
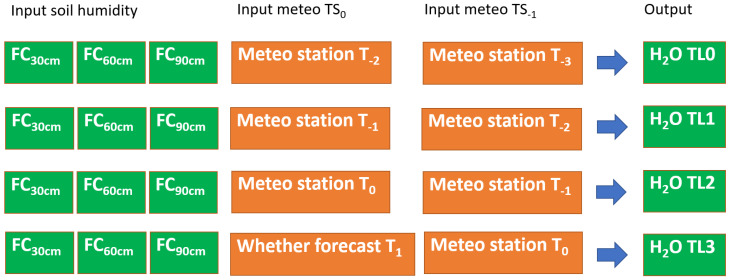
Time-lag, time-series (TS) approach scheme in the application phase; soil moisture at different depths (30, 60, and 90 cm) was substituted with the field capacity at the same depth. The output is represented by the irrigation water forecasted from 0 (TL0; present date) to 3 (TL3) days after.

The commercial ground sensors measured soil temperature and moisture at different depths of 20, 30, 40, 50, 60, 70, 80, and 90 cm (Figure 8). Meanwhile, the open source soil temperature was measured at 30 cm, and the soil moisture LoRa at 30, 60, and 90 cm. Additionally, the irrigation water input estimated indirectly from the consumption in kWh of the pump was added to these data.

The pump of the consortium consumed 900 kWh equivalent to 5.25 mm m^−2^. Table 4 shows the parameters related to the soil structure at different depths.

The optimum moisture (%) for the crop (at different depths) must lie between the values of available water capacity (AWC) and those of field capacity (FC). Above the FC values, the water is dispersed into the subsoil. The objective of the models is to suggest a correct water supply to keep the crop always in the optimum range without going to waste water-soluble fertilizers and/or energy.

In the first phase, the dynamics of the water in the soil at the different layers were studied. To observe the correlation between the input water (rain + irrigation) and the soil moisture at different depths (i.e., 30, 60, and 90 cm), a cross-correlation analysis (Davis, 1986) was carried out on the two columns of daily sampled temporal data. The correlation values at different time (day) lags were calculated, together with the *p* values indicating the significance of the correlation. Cross-correlation analysis was carried out with the software PAST (version 2.17v) [15].

The precision irrigation model was based on the concept of TimeLag/TimeSeries [16]. TimeLag represents the elapse between the water input and the soil moisture data shifted *i* days before and analyzed with the aforementioned cross-correlation analysis. Furthermore, the possibility was considered that the event could be related to the variable of specific adjacent (*n*) days (TimeSeries). Consequently, the possibility to combine the TimeSeries variables was considered to account for variable weights different from the initial condition.

The input block (X-block) in the training phase was constituted by the parameters (daily mean, minimum, and maximum) collected by the weather station (i.e., air humidity (%), air temperature (i.e., maximum, minimum, and mean; °C), wind direction, mean wind speed (km∙h^−1^), rainfall height (i.e., H_2_O rain (mm)), by the parameters measured by the ground sensor (i.e., daily mean soil moisture at different depths (30, 60, and 90 cm)). The Y-block was constituted by the irrigation water input (mm).

The partial least squares (PLS) procedure was elaborated using the PLS Toolbox in MATLAB V7.0 R14 (Math Works, Natick, MA, USA), and included the following steps: (i) the extraction of the dataset (X-block variables); (ii) the creation of a measured values dataset to be used as a reference or response variable (Y-variable); (iii) the data fusion of the two datasets (Y- and X-block) in one analysis dataset (AD); (iv) an analysis dataset partitioning into the model set (MS = 80% of AD) and external validation test set (TS = 20% of ADs) by means of a sample set partitioning based on the joint x–y distances (SPXY) algorithm. This method employs a partitioning algorithm that considers the variability in both x- and y-spaces; (v) application of different preprocessing algorithms to the X- and Y-block (none, Log 1/R, diff1, mean center, autoscale, median center, baseline)—the matrices were preprocessed using the autoscale MATLAB algorithm; (vi) application of chemometric technique—modeling and testing; and (vii) calculation of the efficiency parameter of prediction. Partial least squares consider internal cross-validation of the model set, and we introduced a further validation using the test set. The performances of the model were estimated by evaluating the coefficient of correlation (r) between observed and predicted values, the standard error of prediction (SEP), root-mean-square error of calibration (RMSEC), and bias calculated as the average of the differences between predicted and measured values. Residual predictive deviation (RPD), defined as the ratio of the standard deviation of the laboratory-measured (reference) data to the RMSE, was used to verify the accuracy of the model. The model accuracy and precision were evaluated according to the highest r, minimum SEP, maximum RPD, and bias value very close to zero [17].

After the training phase, the model resulting to be the most efficient and robust was adopted (application phase) for the in-field application and inserted in the web framework for scheduled previsions. The model predicts the irrigation water needs for the same day of the interrogation (T0), for the next day after the interrogation (T1), and the following second and third days after the interrogation (T2, T3). The prevision model replaced the soil moisture at the different depths (30, 60, 90 cm) with the field capacity at the same depths (Figure 8). The open source weather forecast was implemented at TL > 0 using the service Open Weather Map. A scheme of the application phase at different timing is shown in Figure 8.

## 3. Results and Discussion

Results of the cross-correlation analysis for Tenna show highly significant correlation of input water (rain + irrigation) and the soil moisture at 30 cm at time (day) lag 1 (*r* = 0.55845; *p* = 1.31 × 10^−7^). Additionally, at 60 cm, a highly significant correlation was observed at time (day) lag 2 (*r* = 0.46996; *p* = 1.84 × 10^−5^). At 90 cm, no significant correlations were observed. The results show that soil moisture increase after one day at 30 cm depth from the water input on the surface, and after two days at 60 cm. At 90 cm, maybe stochastic influences arise. In Table 5, cross-correlation and *p* values at different soil depths (30, 60, and 90 cm) and time (day) lag from 0 to 5 are reported.

In the next phase, to build the model and predict the irrigation water input, soil moisture at 30 cm was used at time lag −1 (the day before), and soil moisture at 60 and 90 cm were used at time lag −2 (two days before). The best performing and robust model resulted to be time series 2 (two days used), characterized by eight Latent Vectors and an autoscale preprocessing. The performances of the model are listed in Table 6. The model showed good performances having low errors (RMSEC, RMSECV, SEP) and high r values (0.86 in the model and 0.91 in the internal test). The model appeared to be fairly robust (low RPDRMSE values) due to the limited number of input records.

In the operative field condition, the consortium, in the dry season (from the end of April to the end of September), uses to irrigate each parcel every day for 1 h (equivalent to 5.25 mm H_2_O per square meter), rainy days excluded. Considering the period of 78 days of the dry season (from 19 July to 4 September 2019) the Consorzio di Miglioramento Fondiario di Tenna consumed a total of 286 mm of water equivalent to 49,500 kW. The model a posteriori applied on the same period reported a total saving of 255 mm equivalent to 44,000 kW, representing 10.82%.

**Figure 9:**
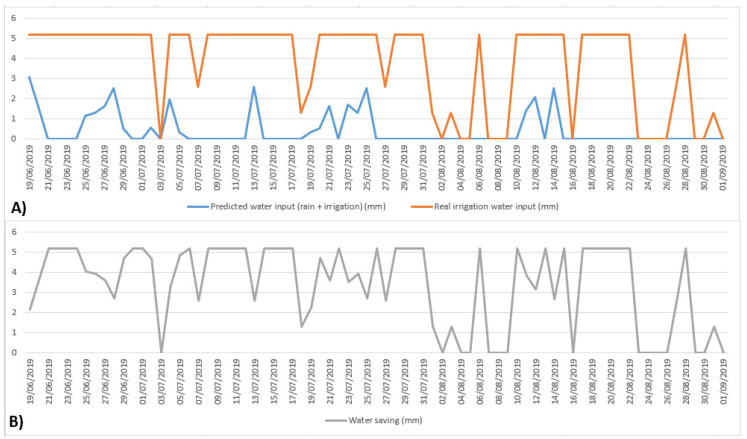
(**A**) Daily irrigation comparison between the partial least squares (PLS) model predicted (proposed) and the real irrigation input water (mm); (**B**) the water-saving comparison (mm).

Figure 9 shows the daily irrigation comparison between the PLS model predicted (proposed) and the real irrigation input water (A), and the water-saving comparison (B). It is possible to observe how the proposed model significantly reduces the irrigation water input.

During the experiment, soil moisture increased after one day from the water input at 30 cm depth, and after two days at 60 cm; this may be attributed to the redistribution of water along the soil profile. At 90 cm, no significant effect was observed. Considering that the two varieties of apple trees are grafted on rootstock M9, which limits the root depth at 40 cm, the water supplies can be considered adequate to the needs of the soil–plant system.

Additionally, in other research studies, the sensors situated at 30 cm depth responded rapidly to irrigation, with a clear amplitude response between minimum pre-irrigation values and maximum terminating irrigation values [9]. In addition, the study by Paris et al. [18] reports a strong relationship between soil moisture profiles and the polydispersity index, indicating an efficient scheduling of the irrigation monitoring soil moisture at 30 and 40 cm of depth.

The rapid response of the system to changes in soil water status avoided water stress during the more sensitive phenologic apple tree phases.

Irrigation strategies based on controlled depletion of soil available water allow us to obtain efficient results in terms of watering depth and number of irrigations for the season, reducing losses by deep percolation [12].

Finally, the forecasting model, applied for 78 days of the dry season, reported a total saving of 255 mm equivalent to 44,000 kW.

The use of the water balance method allows a rapid response to input or output changes, adapting the amount of water to soil–plant system request. Soil water content sensors provided feedback with adequate precision [11,19].

Other authors compared automated scheduling of two independent irrigation plots, differenced by apple trees size (cv. Golden Reinders), with two control plots, the same type of plants, and scheduled manually following a classical water balance. In the plot with automated scheduling irrigation, based on a sensor survey and cultivated with smaller plants, a 24% reduction in water consumption was observed [20].

Nowadays, extremely few contributions document water management variable rate strategies in Italy. Among these, Ortuani et al. [21] experimented a Variable Rate (VR) drip irrigation in northern Italy, achieving a reduction of water input equal to 18% in comparison to the farmer’s conventional irrigation system. This turned out to be effective also in terms of the final product quality producing more homogeneous grape maturation and the same yield.

Although Domínguez-Niño et al. [20] developed an irrigation automated system to manage an apple orchard, the system does not include a remote mobile app to monitor the orchard heterogeneity. Agricultural digitalization represents potential tools to help decision making be the core of DSS.

One of the contributions of the present work, along with the advanced modeling, is data collection and management. Indeed, the developed system collects the data and sends them to the cloud through an IoT structure for remote management of the orchard to lower the energy consumption. One of the main advantages of this smart system is the possibility, in addition to the real-time monitoring, to have the historical data always updated and visible with the possibility to inform watering routines and modify watering schedules in order to improve efficiency.

## 4. Conclusions

The emergence of autonomous irrigation systems based on a variety of technologies is nowadays trying to streamline the orchard’s management while reducing cost and resource use, thus with a positive impact on the environment. The use of IoT technology is becoming a popular choice since it enables farmers to make a decision based on ready-to-use information for pinpointed irrigation decisions, helping them to optimize the crop yield. Unfortunately, many of these have a rigid functioning, present poor integration among platforms, or have prohibitive costs.

In this scenario, the present work integrates different strengths such as low-cost but reliable electronic parts (which is easily replaceable); well-known, convenient, long-range, and low-energy consumption connection protocol (which is LoRaWAN); a flexible design with the possibility to integrate function, data coming from additional sensors, etc.; a user-friendly dashboard and innovative highly performing self-adaptive algorithms.

The results are encouraging in relation to the saving percentages found in the literature, as discussed above.

Furthermore, the economic advantage of using this system opens up excellent opportunities for expanding the research field, making it also generalizable in other contexts.

Future studies, following the potentially integrable system’s characteristics, could point to the development and integration, through the introduction of additional sensors, of precision fertigation strategies for improved apple orchard productivity.

## Figures and Tables

**Table 1:** Climate characterization [i.e., average, minimum, and maximum temperature (°C) and rain (mm)] of Tenna (average of the period 1982–2012). Source: Climate-Data. https://it.climate-data.org/europa/italia/trentino-alto-adige/tenna-113046/#climate-table (accessed on 1 December 2020).

	Jan	Feb	Mar	Apr	May	Jun	Jul	Aug	Sep	Oct	Nov	Dec
Avg T (°C)	−0.6	1.9	6.2	10.8	14.7	18.3	20.7	19.9	16.9	10.5	5.1	0.8
Min T (°C)	−4.1	−2.3	1.4	5.4	9.3	12.6	14.6	14.1	11.5	6	1.5	−2.2
Max T (°C)	2.9	6.2	11.1	16.2	20.1	24.1	26.8	25.7	22.3	15	8.8	3.9
Rain (mm)	40	42	53	69	82	92	84	96	79	85	89	51

**Table 2:** Average monthly temperatures (i.e., minimum and maximum) of 2019 (Source: 3bmeteo).

	Jan	Feb	Mar	Apr	May	Jun	Jul	Aug	Sep	Oct	Nov	Dec
Min T (°C)	−0.6	3.18	4.19	6.71	8.1	16.85	17.07	17.35	13.75	10.59	4.4	2.08
Max T (°C)	6.3	11.48	14.13	13.93	15.71	29.21	28.78	28.03	22.35	17.58	8.92	7.97

**Table 3:** Coordinates of Tenna’s weather stations.

Weather Station	Latitude	Longitude
East Tenna	46,022,687	11,265,643
West Tenna	46,021,743	11,255,331

**Table 4:** Parameters related to the soil structure (organic matter (OM), field capacity (FC), withering point (WP), available water capacity (AWC), density, and total limestone) at different depths (i.e., 30, 60, and 90 cm).

Depth Code	Textural Class	OM (%)	FC (%)	WP (%)	AWC (%)	Density (g cm^−3^)	Total Limestone (g kg^−1^ CaCO^3^)
T1A30_cm_	Sandy loam	1.6	17.1	6.3	10.8	20.54	n.q. (<10)
T1B60_cm_	Sandy loam	1.4	21.6	11.8	9.8	34.56	n.q. (<10)
T1C90_cm_	Sandy loam	0.8	16.0	4.1	11.9	18.81	n.q. (<10)

**Table 5:** Correlation between the input water (rain + irrigation) and the soil moisture at different depths (30, 60, and 90 cm). Cross-correlation and *p* values at different time (day) lag and soil depths. In bold, highly significant correlation values.

Lag	30 cm		60 cm		90 cm	
	Correlation	*p*	Correlation	*p*	Correlation	*p*
**0**	0.27489	0.014864	0.078903	0.49229	−0.015345	0.89392
**1**	**0.55845**	**1.31 × 10^−7−^**	0.37812	0.00069749	0.10545	0.36136
**2**	0.45216	4.12 × 10^−5^	**0.46996**	**1.84 × 10^−5^**	0.13345	0.25044
**3**	0.29893	0.0091823	0.38455	0.0006583	0.10924	0.35084
**4**	0.20259	0.083435	0.31005	0.00718	0.11708	0.3205
**5**	0.23883	0.041858	0.31937	0.005885	0.13446	0.25674

**Table 6:** Characteristics and principal results of the partial least squares regression model to predict the irrigation water input. LVs, latent vectors; RMSEC, root-mean-square error of calibration; RMSECV, root-mean-square error of cross-validation.

**No. of Samples**	31
**Reprocessing X-block**	Autoscale
**No. of LVs**	8
**RMSEC**	2.03
**RMSECV**	6.30
**Bias**	−1.7
**SEP-model**	1.12
**SEP-test**	1.34
**RPD_RMSE_-model**	1.05
**RPD_RMSE_-test**	1.11
**r model (80%)**	0.86
**r test (20%)**	0.91

## Data Availability

Data available on request due to privacy restrictions.
